# Transcatheter occlusion of giant congenital coronary cameral fistulae: a case series

**DOI:** 10.1186/s13256-019-2254-x

**Published:** 2019-10-10

**Authors:** Deogratias A. Nkya, Greenwood Sinyangwe, Farirai Fani Takawira

**Affiliations:** Paediatric Cardiology Unit, Department of Paediatrics and Child Health, Faculty of Health Sciences, University of Pretoria, Bridge C, Steve Biko Academic Hospital, Steve Biko Road, Gezina, Private Bag X323, Pretoria, 0007 South Africa

**Keywords:** Giant congenital coronary cameral fistula, Transcatheter occlusion

## Abstract

**Background:**

A coronary cameral fistula is a rare connection between a coronary artery and a cardiac chamber or vein bypassing the cardiac capillary bed system. Most of these fistulae are congenital and solitary, although they can be acquired and multiple.

**Cases presentation:**

***Case 1:*** A 10-year-old black South African boy presented with a long-standing history of fatigue; he had a heart murmur, and a bounding pulse and wide pulse pressure. An echocardiogram demonstrated a large coronary cameral fistula involving his left coronary artery and his left ventricle. This was also confirmed on ascending aortogram. Surgical ligation was done and his symptoms improved afterward, but a small residual fistula remained. ***Case 2:*** A 7-year-old black South African boy had decreased effort tolerance and a heart murmur on the mid-sternal border. He had cardiomegaly on chest roentgenogram and a dilated left coronary artery origin on echocardiogram. An ascending aortogram confirmed a large left coronary cameral fistula draining to the left ventricle. ***Case 3:*** A 28-year-old black South African woman with decreased effort tolerance and chest pain on exertion had a continuous murmur over the lower sternal border. Echocardiography demonstrated a dilated right coronary artery with a fistulous connection to her right ventricle. An ascending aortogram demonstrated a tortuous coronary cameral fistula arising from her right coronary artery to her right ventricle. All three patients were successfully treated percutaneously using the Amplatzer vascular plug type II device.

**Conclusion:**

The availability of numerous vascular closure devices has made transcatheter occlusion the treatment of choice for the majority of coronary cameral fistulae, rather than the traditional surgical ligation.

## Background

Coronary artery fistulae (CAF) are rare abnormalities of the coronary artery termination, which involve a communication between a coronary artery and either a heart chamber, which is termed as a coronary cameral fistula (CCF), or the arteriovenous system, which is termed as a coronary arteriovenous fistula, bypassing the myocardial capillary bed [1, 2]. The first case was reported in 1865 by Krause *et al.* [3, 4]. The estimated prevalence is 2 per 1000 people [3–6]. The majority of cases are congenital and solitary, and constitute 0.2–0.4% of all congenital cardiac malformations and 14% of all coronary anomalies [7–9]. The embryology of CAF is suggested to be the persistence of sinusoidal connections [10]. The majority (60%) of the fistulae arise from the right coronary artery (RCA). Out of all CAF, 90% drain to the low-pressure right heart chambers: 41% in the right ventricle (RV), 26% in the right atrium (RA), 17% in the pulmonary arteries, and 6% in the caval veins. Only 10% drain to the left ventricle (LV) and left atrium (LA) [11]. We describe three patients with giant congenital CCF, which were successfully occluded transcutaneously using the Amplatzer vascular plug type II (AVP II; St Jude Medical, St Paul, MN, USA).

## Cases presentation

### Case 1

**Fig. 1:**
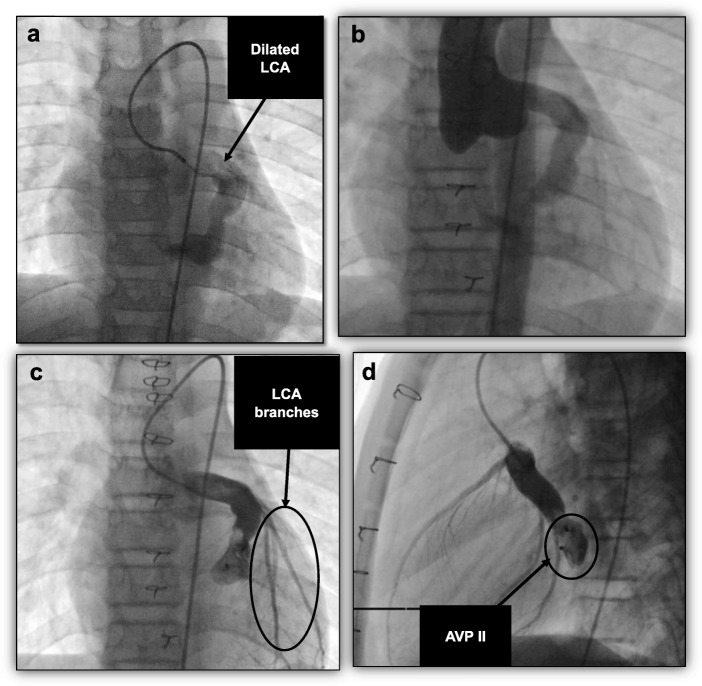
Case 1. **a** Selective LCA angiogram in anteroposterior view, showing a dilated LCA with a large fistulous connection to the LV before surgical ligation. **b** An ascending aortogram in anteroposterior view showing residual fistula from the dilated LCA to the LV after surgical ligation. Selective LCA angiograms in anteroposterior (**c**) and lateral (**d**) views showing the deployed AVP II with complete occlusion of the residual fistula. *AVP II* Amplatzer vascular plug type II, *LCA* left coronary artery, *LV* left ventricle

A 10-year-old boy black South African presented with a history of easy fatigue on exertion. There was no past history of recurrent illness or hospital admission. There was no history of congenital heart diseases in his family. A clinical examination on admission revealed a well-grown boy weighing 26.6 kg. He had no signs of respiratory distress or heart failure. His body temperature was 37 °C. He had a bounding pulse with a rate of 100 beats per minute. His blood pressure (BP) was 100/20 mmHg, demonstrating a wide pulse pressure. A grade 3/6 continuous murmur could be heard at the fourth left intercostal space. The rest of his systemic and neurological examination was normal. He had normal posture, gait, muscle bulkiness, tone, power, and reflexes. There was no hepatosplenomegaly or jaundice. His complete blood count showed hemoglobin (Hb) of 12.2 g/dl, white blood cell count (WBC) of 8.7 cells/mm^3^ with normal differential counts, and a platelet count of 445 cells/mm^3^. His C-reactive protein (CRP) was not elevated (< 1 mg/dl). He had a normal creatinine of 55 μmol/L and urea of 3.8 mmol/L. His serum electrolytes were also normal (sodium, 138 mmol/L; potassium, 3.9 mmol/L; and chloride, 100 mmol/L). A chest roentgenogram and electrocardiogram (ECG) were normal. Echocardiography demonstrated a large fistula involving his left coronary artery (LCA) and his LV. The LCA origin was massively dilated and measured 10 mm. An ascending aortogram confirmed a diagnosis of a large CCF from the LCA to the LV (Fig. 1a). His RCA appeared normal.

After discussion with the surgical team, it was concluded that the fistula would be too big for successful deployment of a device without the risk of embolization. Surgical ligation of the fistula was then elected and performed. Post-surgical ligation echocardiography demonstrated residual flow through the fistula into the LV, but the flow was minimal and restrictive with a gradient of 80 mmHg. His symptoms resolved. He was monitored over a period of 2 years and remained asymptomatic, but the residual fistula remained. Due to the risk it posed for infective endocarditis, we elected to close the residual fistula percutaneously. A repeat angiogram into the ascending aorta and LCA demonstrated a fistula with its narrowest portion at the site of surgical ligation measuring 8 mm (Fig. 1b). His LCA was cannulated using a left Judkins catheter and a V18 guide wire. A 16 mm AVP II was successfully deployed distally at the site of previous surgical ligation. Post device closure, an angiogram showed no flow through or around the device and there was better flow into the left anterior descending (LAD) and circumflex arteries (Fig. 1c, d). He was discharged and allowed to go home the following day on 37.5 mg clopidogrel daily and 100 mg aspirin daily. These medications were stopped after 6 months. He has remained well 10 years after the procedure.

### Case 2

**Fig. 2:**
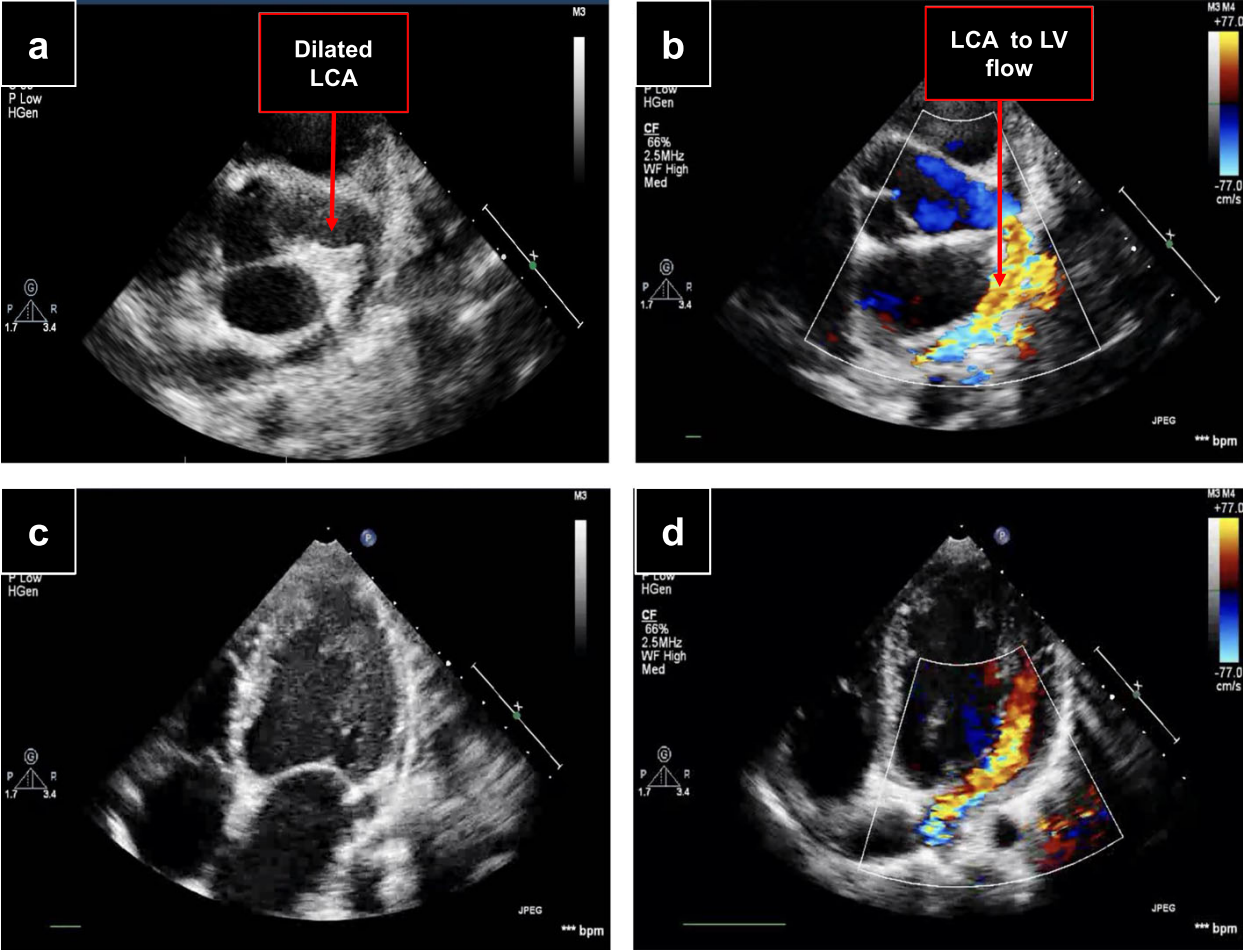
Echocardiogram images of case 2. Parasternal short-axis views showing a dilated LCA origin (**a**) with color flow Doppler (**b**). Apical four-chamber views showing dilated LV (**c**) and color flow Doppler showing fistulous flow entering the dilated LV (**d**). *LCA* left coronary artery, *LV* left ventricle

A 7-year-old black South African boy was found to have an incidental murmur during evaluation for an upper respiratory tract infection. On direct questioning, he admitted having decreased effort tolerance. There was no significant past medical history and he was not receiving any chronic medication. A clinical examination revealed a healthy child with no fever. He had normal BP of 100/65 mmHg, a bounding pulse and a rate of 120 beats per minute. There was mild cardiomegaly. A grade 3/6 continuous machine-like murmur was detected on the mid-sternal border. He had normal chest symmetry. There was no liver or spleen enlargement. A neurological examination was normal. Basic blood investigations were within normal limits as follows: Hb, 11.6 g/dl; WBC, 10 cells/mm^3^; platelet count, 332 cells/mm^3^; serum creatinine, 48 μmol/L; urea, 3.7 mmol/L; sodium, 130 mmol/L; potassium, 4.8 mmol/L; and chloride, 98 mmol/L. Chest radiography confirmed cardiomegaly. An ECG was found to be normal, with no signs of ischemia. Two-dimensional echocardiography with color flow Doppler showed a grossly dilated LCA origin measuring 10 mm (Fig. 2a, b) and draining into the dilated LV (Fig. 2c, d). A diagnosis of a large CCF from the LCA to the LV was made. His LV function was normal with an ejection fraction of 63%.

**Fig. 3:**
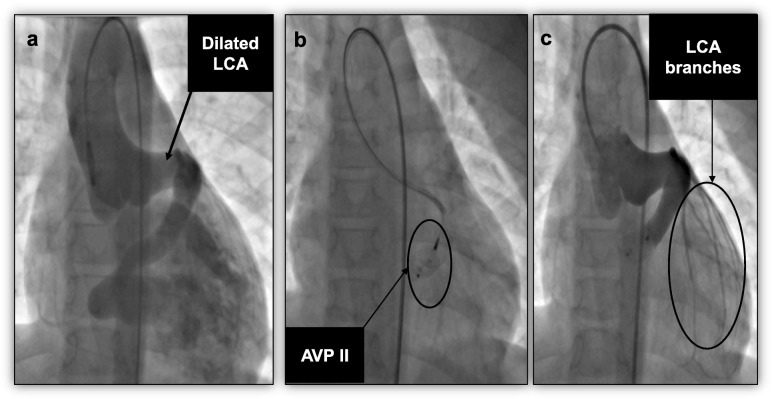
Case 2 angiography in anteroposterior view. **a** An ascending aortogram showing a dilated LCA origin with a large coronary cameral fistula to the LV. **b** Fluoroscopy showing an AVP II that has been deployed to the fistula, still attached to the delivery cable. **c** A selective LCA angiogram demonstrating complete occlusion of the fistula by the AVP II. *AVP II* Amplatzer vascular plug type II, *LCA* left coronary artery, *LV* left ventricle

At cardiac catheterization, an ascending aortogram confirmed the diagnosis of a large CCF draining to the LV (Fig. 3a)*.* After the angiogram, we elected to close the fistula percutaneously using the AVP II device. The LCA was cannulated using a left Judkins catheter. A 14 mm AVP II was deployed into the exit end of the fistula into the LV, to avoid occlusion of the major LCA branches (Fig. 3b). A repeat angiogram showed successful device closure of the fistula, with no residual leak around or through the device on angiogram (Fig. 3c). A post closure echocardiogram showed no residual fistulous flow and he had normal cardiac function. He was discharged on 75 mg aspirin daily, which was stopped after 6 months. On subsequent reviews, he has remained well and asymptomatic. There is no recanalization of the fistula 7 years after the procedure.

### Case 3

A 28-year-old black South African woman presented with a history of decreased effort tolerance and episodes of chest pain on exertion. She reported that a cardiac murmur had been heard during her childhood, but it had never been investigated. She had never smoked tobacco or consumed alcohol. On examination, she had New York Heart Association (NYHA) class II cardiac failure. She had no fever or jaundice. Her pulse was 75 beats/minute and collapsing. She had a bounding radial and femoral pulse. Her BP was 120/50 mmHg, with a wide pulse pressure. There was no cardiomegaly and no evidence of pulmonary hypertension. A grade 2/6 continuous murmur was audible over the left lower sternal border. She had no liver or spleen enlargement. A central nervous system examination was unremarkable with normal cognition, normal muscle power, bulkiness, and gait. She had no lateralizing signs and had normal vision. Her chest roentgenogram and ECG were normal. There were no features of ischemic heart disease evident from the ECG. Echocardiography demonstrated a dilated RCA origin that formed a large fistulous connection with the RV. Her LCA was normal. She had normal left ventricular function. Hemodynamic data demonstrated a left-to-right shunt with a pulmonary to systemic blood flow ratio (Qp:Qs) of 1.3:1. The pulmonary pressure and pulmonary vascular resistance were normal.

**Fig. 4:**
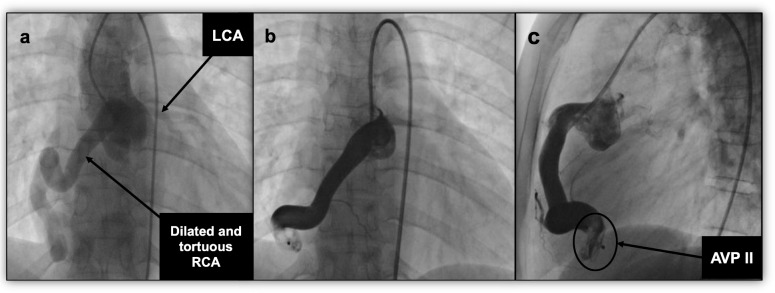
Case 3 Angiography. **a** An ascending aortogram demonstrating a dilated RCA with a tortuous fistulous connection to the RV. Selective RCA angiograms in anteroposterior (**b**) and lateral (**c**) views demonstrating the AVP II with complete occlusion of the fistula. *AVP II* Amplatzer vascular plug type II, *LCA* left coronary artery, *RCA* right coronary artery, *RV* right ventricle

The angiography of the ascending aorta and RCA demonstrated a large and tortuous CCF arising from the RCA and draining into the RV. The fistula measured 11 mm at the widest point and 8.5 mm at the narrowest point (Fig. 4a). The fistula was easily accessed from the RCA with a 6F-guiding catheter, which could be manipulated into the distal part of the fistula with the aid of a 0.014-inch coronary guide wire. A 14 mm AVP II was selected and deployed into the distal portion of the fistula. The position was checked with angiography, which confirmed that there was no obstruction of coronary side branches. There was also no residual shunt present across the AVP II. Repeat angiography showed complete occlusion of the fistula and better perfusion to the RCA and its branches (Fig. 4b, c).

Twelve hours after the procedure, our patient complained of chest pain. An ECG was done; it was normal with no evidence of ischemia. Her cardiac enzymes were also normal. Her chest pain resolved after the administration of ibuprofen. Repeat echocardiography confirmed the patency of the RCA, and occlusion of the fistula. She developed mild aortic regurgitation and her left ventricular function was marginally depressed with an ejection fraction of 55%. These new developments were thought to be due to elevated systemic vascular resistance following the removal of the left-to-right shunt and run-off into the RV.

She was discharged 48 hours after the procedure on 5 mg/kg aspirin daily to prevent thrombosis in the residual cul-de-sac. On follow up at 1 month and 6 months after the procedure, her pre-occlusion symptoms and her chest pain had resolved. The aortic regurgitation had resolved, and her left ventricular function had normalized. She remains symptom free to date, following the occlusion of the CCF.

## Discussion

We have presented three cases of giant CCF. Two cases involved the LCA draining into the LV and one case of the RCA draining to the RV. All three patients presented with a history of easy fatigue on exertion and the 28-year-old woman had associated chest pain, as well as fatigue. Challoumas *et al.* and Cheung *et al.* described fatigue and chest pain on exertion as the most common symptoms [1, 3]. The most common signs were a continuous heart murmur and cardiomegaly [1].

The pathophysiology of CCF is dependent on the size and the site of termination. Small fistulae are usually asymptomatic and are often found incidentally on echocardiography or angiography done for an unrelated cause. Large fistulae cause symptoms such as high output cardiac failure, ischemia, or mural thrombus formation [2, 7, 12]. The fistulae in our patients were large and all patients were symptomatic.

Traditionally, small asymptomatic fistulae were managed conservatively with no surgical intervention due to their benign nature and the possibility of spontaneous closure. Medium-sized and large-sized fistulae were managed by surgical ligation [2]. Reviews done by Mavroudis *et al.* and Cheung *et al.* showed that surgical ligation was a safe procedure, with a 10% recurrence rate [1, 13].

A residual fistula was documented in our 10-year-old patient, who had to undergo percutaneous transcatheter closure. Surgical ligation involves placing an external suture around the fistula. Should the surgical knot not be tight enough, the lumen may not be completely occluded, resulting in a residual fistula. Unlike percutaneous closure, where angiography is used to cross check and confirm successful occlusion, there is no similar intraoperative tool for the surgeons, which sometimes results in residual fistulous flow. The American College of Cardiology/American Heart Association (ACC/AHA) Guidelines for the Management of Adults with Congenital Heart Disease recommends closure of large fistulae regardless of symptomatology [14].

Reidy *et al.* performed the first successful transcatheter closure of a large coronary to bronchial fistula using a detachable balloon [2, 15]. Amplatzer duct occluders and vascular plugs have made it possible to successfully occlude large fistulae using a single device [2]. The three patients we have described are examples of large CCF that were successfully occluded using the AVP II without residual flow. Other devices include coils, a double umbrella device, and covered stents, which can be used for small-sized and medium-sized fistulae.

Transcatheter occlusion for CCF is indicated when the anatomy of the fistula is favorable, in order to avoid complications associated with the procedure [3]. These include ventricular arrhythmia, coronary spasm, coronary perforation or fistula dissection, device embolization, and clot formation around the device [4, 6, 11]. There have been reports of thrombus formation in the residual large cul-de-sac, proximal to the deployed device, leading to occlusion of the coronary artery and myocardial infarction [16].

Contraindications to transcatheter occlusion of CCF include multiple fistulae communications, tortuous fistulae, and the presence of other cardiac anomalies that would require surgical correction [2, 11]. Our patients did not develop any complications during or after the procedure or on long-term follow up. Surgery is still a recommended intervention for all types of fistulae, but it has several disadvantages over percutaneous transcatheter closure. These are sternotomy wound and associated healing complications, wound infection, post pericardiotomy syndrome, bleeding, cardiopulmonary bypass (CPB) and its associated complications, and prolonged hospital stay after the operation [2, 3].

There are no randomized clinical trials that have compared treatment outcomes and complications on transcatheter closure versus surgical ligation of CCF, because this is a rare condition, which makes it difficult to get adequate numbers for an appropriate randomized clinical trial. Armsby *et al.* compared their cohort of 33 patients who had successful transcatheter occlusion of CCF with published surgical reports and found no differences in outcomes and complications between the two techniques [17]. However, they did not discuss the social impact of the permanent surgical sternotomy scar and the length of hospital stay, which are obvious differences between surgical ligation and transcatheter closure of CCF [16, 17].

The ACC/AHA Guidelines recommend both surgical and transcatheter interventions for management of CCF, and the criteria for treatment are the same for both interventions. Transcatheter intervention is recommended over surgery, if the fistula is favorable for this intervention modality [14].

## Conclusion

Transcatheter occlusion of CCF is now considered the treatment of choice for most fistulae. Surgical ligation is reserved for complicated fistulae. Newer devices, such as the Amplatzer vascular plug (AVP), have broadened the scope of management of large CCF, which were previously not suitable for device closure.

## Data Availability

The angiogram images are saved in the picture archiving and communication system (PACS) of our hospital. The echocardiogram images are archived in CDs at our cardiology clinic. Patient records are archived in the hospital record system. All the data used in these case reports are available upon request.

## Abbreviations

ACC: American College of Cardiology
AHA: American Heart Association
AVP: Amplatzer vascular plug
AVP II: Amplatzer vascular plug type II
BP: Blood pressure
CAF: Coronary artery fistulae
CCF: Coronary cameral fistulae
CPB: Cardiopulmonary bypass
CRP: C-reactive protein
ECG: Electrocardiogram
Hb: Hemoglobin
LA: Left atrium
LAD: Left anterior descending artery
LCA: Left coronary artery
LV: Left ventricle
NYHA: New York Heart Association
Qp:Qs: Pulmonary to systemic blood flow ratio
RA: Right atrium
RCA: Right coronary artery
RV: Right ventricle
WBC: White blood cell count
